# Subcritical Water Treatment for Valorization of the Red Algae Residue after Agar Extraction: Scale-Up from Laboratory to Pilot Plant

**DOI:** 10.1021/acs.iecr.2c04132

**Published:** 2023-02-16

**Authors:** Esther Trigueros, Cipriano Ramos, Patricia Alonso-Riaño, Sagrario Beltrán, María Teresa Sanz

**Affiliations:** Department of Biotechnology and Food Science, Chemical Engineering Division, University of Burgos, Plza. Misael Bañuelos s/n, Burgos 09001, Spain

## Abstract

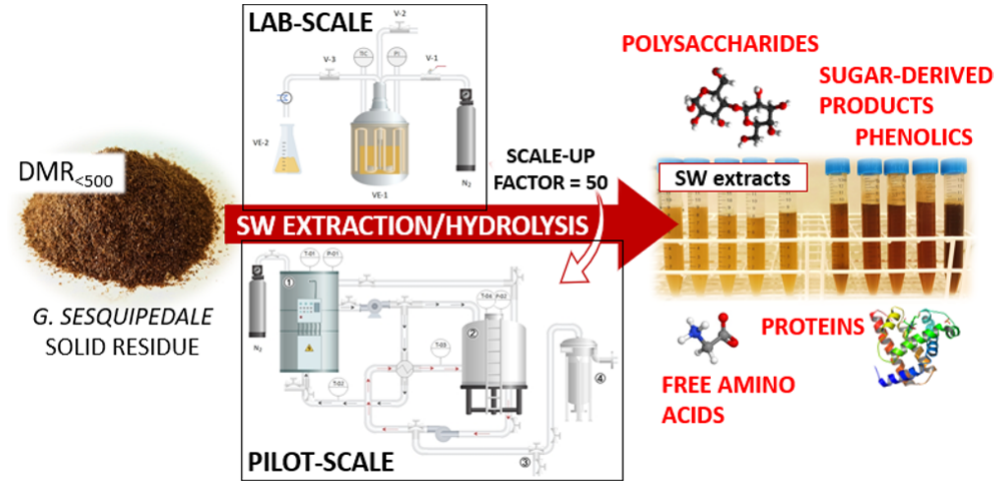

The feasibility of industrial subcritical water treatment on *Gelidium sesquipedale* residue through scaling up from the lab to pilot system in discontinuous mode (geometric scale-up factor = 50), at 130 and 175 °C (5% biomass), was investigated. The maximum volumes of the reactors were 500 mL at the lab-scale and 5 L at the pilot-scale system. At 175 °C, faster extraction/hydrolysis was observed for the pilot plant, but maximum yields were similar: 71.4 and 78.6% for galactans, 9.8 and 10.4% for glucans, and 92.7 and 86.1% for arabinans in pilot scale and lab scale, respectively, while the yields for proteins accounted nearly 40%. The highest yields for amino acids were observed for the smallest ones, while lower yields were determined for polar amino acids. The total phenolic content and color intensity progressively increased along time at lab scale, while a plateau was reached at the pilot level. Lower extraction yields but reproducible results were obtained at 130 °C. Finally, the pilot scale was essayed at a higher biomass loading (15%), and successful results were obtained, supporting the feasibility of the scaling-up process.

## 1 Introduction

The solid residue obtained from *Gelidium sesquipedale* red alga after industrial agar extraction still contains large amounts of different bioactive compounds. Among them, proteins with all the essential amino acids and carbohydrates such as glucans, galactans, or arabinans stand out.^1^ Despite being generally discarded, its reincorporation in the industry would be possible within a biorefinery concept, which is referred to the production of high-value compounds from biomass by means of green technologies in an economical, efficient, and environmentally friendly way.^2^

Subcritical water (SW) hydrolysis/extraction stands out among green technologies as a great alternative to traditional extraction processes. SW treatment consists of using hot pressurized liquid water above its boiling point, 100 °C, and below its critical point, 374 °C. At these conditions, many properties of water as solvent, such as density, dielectric constant, or its ionic product, change greatly in comparison with the properties of water at room temperature and atmospheric pressure.^3,4^ The dielectric constant of water is related to polarity and decreases with increasing temperature. Specifically, the value drops from 80 at room temperature to 40 at 200 °C, this value being similar to those of the organic solvents. Consequently, through the dielectric constant modulation with temperature, SW is able to selectively extract both polar and nonpolar compounds.^5,6^ Moreover, under subcritical conditions, the ionic product of water increases, and water is highly dissociated into H^+^ and OH^–^ ions, which are available in the reaction medium favoring ionic reactions.^7^

As any other process, SW treatment can be operated in a continuous or discontinuous mode depending on whether there is a constant flow of materials at the inlet and outlet of the process or they are placed into the treatment vessel and allowed to evolve with time. Furthermore, a semicontinuous system is possible with the combination of the two modes above, meaning that biomass is charged into the reactor and freshwater is continuously pumped through the reactor.^8^ Recently, the results obtained after SW extraction/hydrolysis from *G. sesquipedale* residue in semicontinuous mode have been reported. A greater and faster extraction/hydrolysis was observed when the residence time was reduced. As an example, by working at 56.3 min of residence time (185 °C), almost 70% of the protein was recovered;^1^ however, the extraction yield was almost 100% by decreasing the residence time down to 3.0 min at the same temperature.^9^ The same trend was observed for the release of free amino acids and the extraction/hydrolysis of the oligomer fraction (glucans and galactans). However, a discontinuous SW system has not yet been evaluated for this algal residue valorization.

Generally, the design of the industrial SW equipment is preceded by the study of laboratory- and pilot-scale systems, but in many cases, the pilot-scale evaluation stage is avoided and goes directly from the laboratory to the industrial scale. Nevertheless, the scaling-up process would be much more efficient by incorporating the pilot-scale study in order to obtain quality data and determine the scale-up factor.^10^ Hence, in order to assess the viability of the SW industrial treatment for the algal residue valorization, the pilot-scale process should be studied.^11^

Several companies are already working within the context of subcritical water extraction on an industrial scale. For instance, Sensient (USA) works on obtaining extracts from different plant materials such as cocoa, rosemary, or green tea; part of the Celabor (Belgium) activity is aimed to obtain plant flavors from coffee and marine products, soluble vitamins, and phytonutrients, while C2FUT (Italy) is focused on the use of subcritical water for food processing. However, little has been described in the literature about the use of this technology at the industrial level from agri-food industry waste. Cravotto et al.^12^ studied the scale-up process from the laboratory scale to semi-industrial subcritical water extraction system, but only the extraction yield, the polyphenol content, and the antioxidant capacity of the collected extracts were evaluated. Moreover, Thiruvenkadam et al.^13^ analyzed the recent developments in subcritical water extraction scale-up, concluding that, although the extraction by means of subcritical water has been investigated, there is still a commercial interest and a need for the development of these systems.

The main goal of this research is to contribute knowledge about subcritical water treatment at the industrial scale through scaling up from the lab to the pilot system. Accordingly, the purposes of this work are (1) to study the subcritical water ability in a discontinuous mode to recover targeted bioactive compounds from algal residue and (2) to compare and to assess the reproducibility of lab- and pilot-scale subcritical water performances.

## 2 Materials and Methods

### 2.1 Raw Material

The raw material used in this work was kindly provided by Hispanagar (Burgos, Spain) (www.hispanagar.com/es). It consists of the industrial solid residue of the red alga *Gelidium sesquipedale* after industrial agar extraction. Before use, this residue was oven-dried at 45 °C for 24 h to obtain a dried macroalga residue (DMR) with a humidity of 5 ± 2%. This DMR was milled by using a Retsch mill (model SM100, 1.5 kW, 1500 rpm), and the particle size distribution of the material used as the feed of the SW treatment was determined by using different bottom sieves with an aperture size of 125, 250, 500, and 1000 μm (Cisa Sieving Technologies). The DMR particle size distribution is shown in Figure S1. The major fraction (74.6%) was smaller than 500 μm, while just 2.3% was larger than 1000 μm. The DMR fraction below 500 μm, henceforth referred to as DMR_<500_, was used for SW treatment due to the requirements of the recirculation pump used to achieve homogenization in the SW pilot plant vessel. Raw material characterization was carried out according to the NREL protocols (https://www.nrel.gov/bioenergy/biomass-compositional-analysis.html) and is shown in Table 1.

**Table 1:** Chemical Composition of Dried Macroalga Residue (DMR) and DMR Size Lower than 500 μm (DMR_<500_), Expressed as % (w/w) ± Standard Deviation

compound	DMR	DMR_<500_
extractives	11.5 ± 0.9^b^	9.6 ± 0.9^b^
carbohydrates	37 ± 2^c^	33 ± 2^b^
glucans	23.4 ± 0.9^c^	21.4 ± 1.2^b^
galactans	10.9 ± 0.5^b^	10.3 ± 1.7^b^
arabinans	2.9 ± 0.2^c^	1.51 ± 0.11^b^
lignin	12 ± 1^c^	7.6 ± 0.9^b^
soluble	8.7 ± 0.1^b^	7.0 ± 0.9^b^
insoluble	3 ± 1^c^	0.6 ± 0.2^b^
proteins^a^	21 ± 1^c^	17.6 ± 0.5^b^
lipids	0.87 ± 0.09^c^	2.3 ± 0.5^b^
ashes	22 ± 2^c^	24.9 ± 1.0^b^

a Proteins include the protein content in the extractive fraction (DMR: 2.6 ± 0.2%; DMR_<500_: 1.9 ± 0.1%; NF = 4.9). Values with different letters in each column are significantly different when applying Fisher’s least significant difference (LSD) method at *p* value ≤0.05 (*n* = 3 technical replicates).

### 2.2 Subcritical Water Equipment

#### 2.2.1 Laboratory-Scale Equipment and Procedure

Lab-scale SW treatment in a discontinuous mode was performed by using a stainless steel reactor of 500 mL volume. The heating system consisted of a ceramic heating jacket (230 V, 4000 W, Ø95 cm, and 160 mm height) covering the reactor, which allows the system to reach the working temperature (Figure S2a). A Pt100 sensor connected to a PID system and placed inside the reactor allowed to control and register the temperature during the SW treatment.

In a typical run, 17.5 g of DMR_<500_ was charged into the reactor together with 350 mL of deionized water (5% biomass). The mixture was heated up to the desired temperature at a certain heating rate, and pressure was fixed to 50 bar by using nitrogen gas to prevent sample oxidation. Mechanical stirring (500 rpm) was used in order to maintain biomass as a solid suspension. Extraction/hydrolysis kinetics were followed by periodically withdrawing the sample through a sampling pipe submersed in the mixture and provided with a metallic filter to avoid the clogging of the pipe. Laboratory-scale SW extraction/hydrolysis was carried out at 130 and 175 °C for a total treatment time of 130 min.

#### 2.2.2 Pilot-Scale Equipment and Procedure

Subcritical water experiments at the pilot-scale level were carried out at the facilities of the company Hiperbaric S.A. (Burgos, Spain) by using a discontinuous system.

The main structural elements of the prototype were a reactor of 25 L capacity, a steam boiler as the heating system, a recirculation pump to maintain the solid suspension of the biomass inside the reactor, a heat exchanger to avoid cooling during the recirculation process, and a solid/liquid separation system (Figure S2b). Hence, a geometric scale factor of 50 was evaluated in the scaling up study.

The maximum operational specifications of the designed pilot-scale system were 185 °C and 20 bar. Operation and control of the process were performed by the self-built Hiperbaric software.

**Figure 1:**
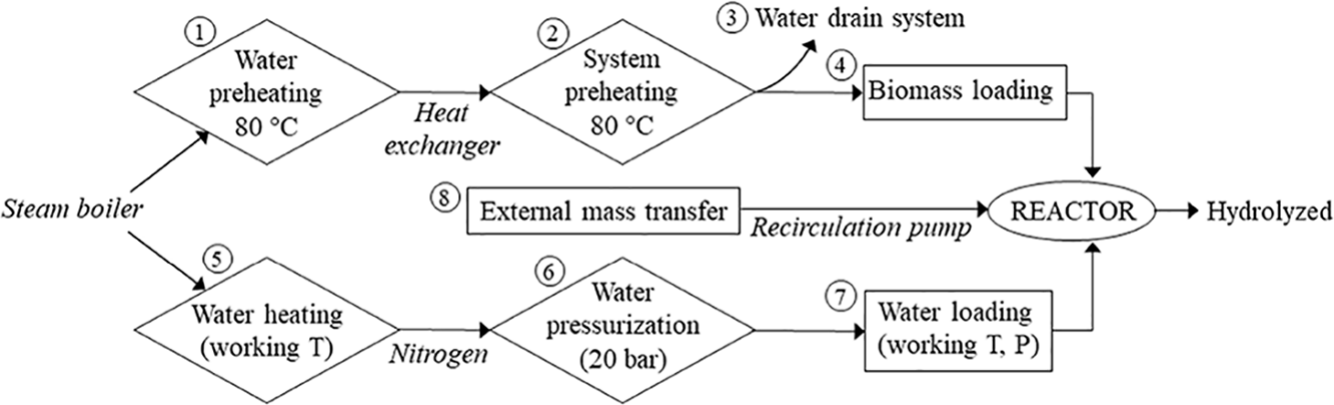
Flowchart of the process carried out before starting the extraction in the subcritical water extraction system on a pilot scale. The numbers in circles represent the sequential order of each stage in the overall process.

In a typical run, water was initially preheated up to 80 °C in the steam boiler and circulated through the heat exchanger. This way, the whole system was initially preheated at this temperature. After this preheating period, the system was completely drained, and the biomass was charged into the reactor. Then, the reactor was filled with pressurized water at the working temperature, achieved by the steam boiler system. The system was pressurized by using nitrogen gas. The recirculation pump was turned on to enhance external mass transfer in the extraction/hydrolysis process. The flowchart of this process is shown in Figure 1. The pump was able to handle up to a biomass concentration of 4 wt % with a maximum particle size of 500 μm, which determined the particle size of the biomass to be used. The pump requirements were a maximum operating pressure of 20 bar and a feed flow rate of 300 L/h. The heat exchanger placed in the recirculation pipe allowed contact with the steam boiler outlet pipe, avoiding cooling in the recirculation process. A sampling system at the bottom of the reactor allowed sample withdrawal to follow the extraction/hydrolysis kinetics. After the completion of the SW treatment, the filtration tank allowed phase separation to obtain a liquid hydrolysate and the solid residue.

In order to assess the feasibility of the subcritical water treatment for the proposed raw material at the pilot level, experiments were carried out at 130 and 175 °C for 130 min with 5 wt % of biomass loading and a working pressure of 20 bar. Finally, once the scaling-up process was evaluated, an experiment using 15% (w/v) of biomass loading was carried out in the pilot-scale reactor for a total treatment time of 76 min in order to compare the concentration of each compound present in the extract with those obtained by using a lower biomass load at the same scale reactor. The treatment time was selected by taking into account the extraction kinetics of the compounds analyzed.

### 2.3 Analytical Methods

#### 2.3.1 Sugars and Derived Compounds

Sugars and the derived compounds were measured by using an HPLC system equipped with a Biorad Aminex-HPC-87 H column, a variable-wavelength detector (VWD), and a refractive index detector (RID), as described by Trigueros et al.^14^ The column and the detectors were maintained at 40 °C, and 0.005 M sulfuric acid was used as the mobile phase with a flow rate of 0.6 mL/min.

Monomeric sugars and sugar-derived compounds were directly measured in liquid extracts after filtering through a 0.22 μm pore size syringe filter (Scharlab). The oligomeric sugar fraction needed to be hydrolyzed in order to release the monomeric sugars for quantification according to the National Laboratory Analytical Procedure (https://www.nrel.gov/bioenergy/biomass-compositional-analysis.html). Briefly, the hydrolysis process involved a first autoclaved acid hydrolysis stage by using 72% w/w sulfuric acid for 1 h at 121 °C, followed by cooling at room temperature, and a final neutralization stage to pH 5–6 with calcium carbonate. Monomeric and total sugar yields were estimated according to:^15^
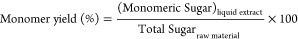
1
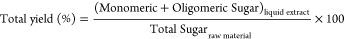
2

Oligomeric sugars were determined as the difference between the total and monomeric sugars in the liquid extracts by applying an anhydro correction factor, and the oligomer yield was evaluated as the ratio between oligomeric and total sugars.

#### 2.3.2 Protein and Free Amino Acids

The total protein content was determined from the nitrogen content in the liquid extracts by applying a nitrogen factor of 4.9 estimated from the amino acid profile of the raw material.^1^ A nitrogen factor of 6.25 is traditionally used to calculate crude protein, assuming that protein is composed by 16% of nitrogen and a negligible nonprotein nitrogen. However, the presence of pigments and inorganic nitrogen in seaweeds makes these values lower due to the nonprotein nitrogen content increase and hence the nitrogen factor reduction.^16^

The nitrogen content was measured by using a TOC/TN analyzer (Shimadzu TOC-V CSN analyzer) using KNO_3_ as the standard.

Free amino acids were determined by using the EZ:faast Phenomenex procedure. Briefly, it consists of a first solid extraction, followed by a derivatization step, and a final liquid/liquid extraction. Then, the derivatized samples were analyzed by gas chromatography (Hewlett-Packard, 6890 series) coupled to an FID.

#### 2.3.3 Total Phenolic Content

Total phenolic content (TPC) was determined by the Folin–Ciocalteu reagent according to Singleton et al.^17^ and expressed as grams of gallic acid equivalent (GAE) per kilogram of DMR_<500_.

#### 2.3.4 Elemental Composition

The elemental composition (C, H, N, and S) of the raw material and solid residues after SW treatment was determined by using an organic elemental micro-analyzer equipment (Thermo Scientific Model Flash 2000). Ash content was estimated by placing around 0.5 g of the sample in a muffle furnace at 575 ± 25 °C for 24 ± 6 h until constant weight. The oxygen content was determined from the difference.

The high heating value (HHV) was evaluated by the following equation:^18^
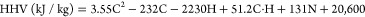
3

### 2.4 Statistical Analysis

All determinations were made at least in duplicate and expressed as mean ± standard deviation. The Fisher’s least significant difference (LSD) method at *p* value ≤0.05 was applied to confirm significant differences. Analyses were carried out by Centurion Statgraphics software.

## 3 Results and Discussion

### 3.1 Raw Material Characterization

The DMR_<500_ composition in comparison with DMR is shown in Table 1. Grinding and sieving processes resulted in a statistically significant reduction of total carbohydrates, proteins, and lignin content in DMR_<500_. After processing, insoluble lignin accounted for just 0.6 ± 0.2%, while this content was 5 times higher in the original sample; therefore, this lignin is supposed to remain mainly in fractions greater than 500 μm, which were around 25% of the initial DMR (see Figure S1). In this sense, grinding and sieving favored sample preconditioning before SW treatment, due to the fact that a lignin removal step is usually needed when working with second-generation biomasses such as agricultural and forest residues with a high lignin content.^19^ Moreover, a slight reduction in carbohydrates and protein content in DMR_<500_ in comparison with original DMR was observed, but still a high content of carbohydrates (33.2 ± 2.1%) and proteins (17.6 ± 0.5%) was found. As a result of this reduction, other compounds such as lipids and ashes increased, remaining in the smallest fractions. However, this raw material (DMR_<500_) is also considered a low-lipid algal biomass, as the lipid fraction continued to be small (2.3 ± 0.5), which could represent an advantage for subcritical water extraction.^13^

### 3.2 Feasibility of the Subcritical Water Treatment: Scaling-Up Study

#### 3.2.1 Heating Rate

**Figure 2:**
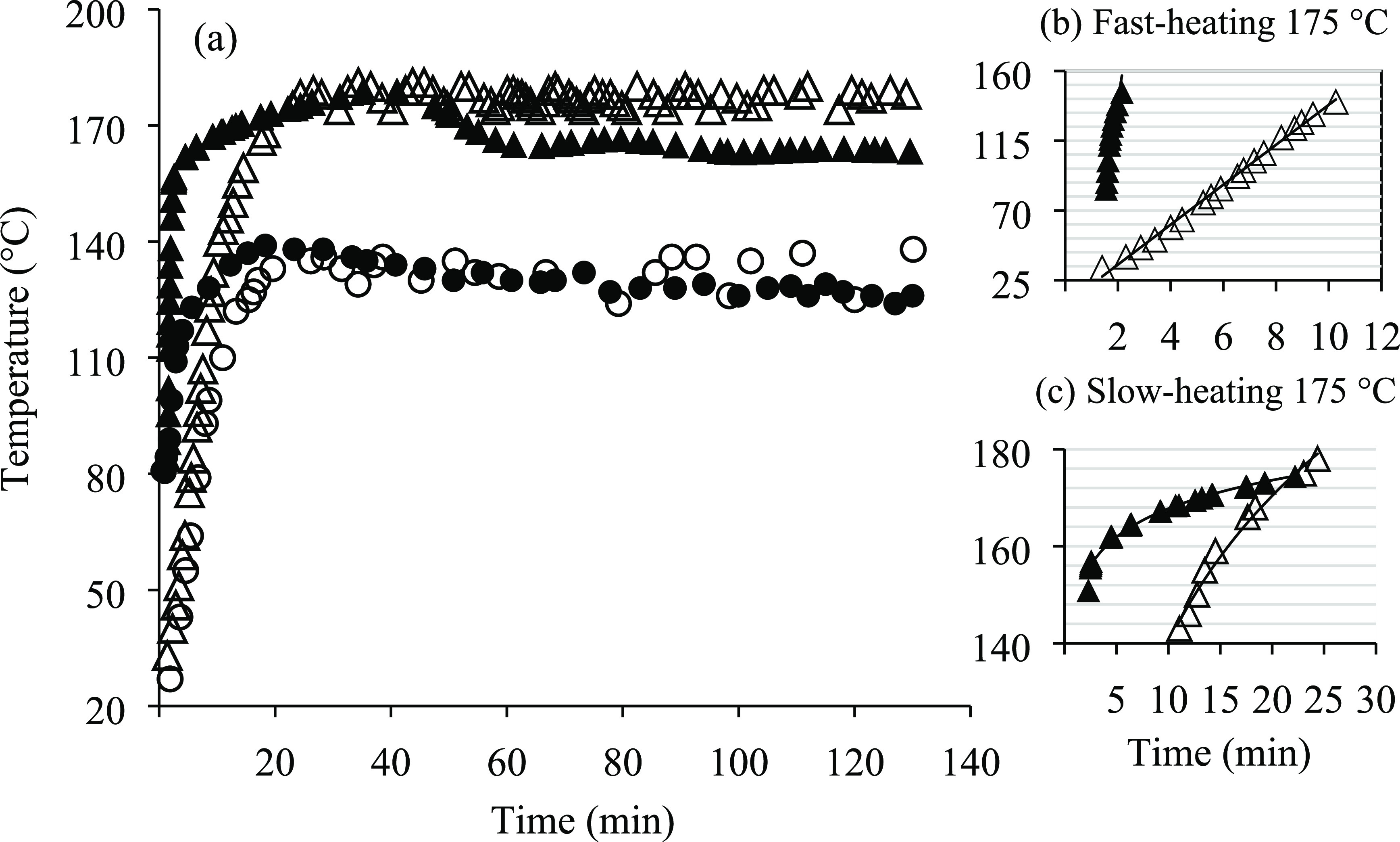
(a) Extraction temperature profiles along the treatment time at lab scale: 130 °C (○) and 175 °C (△); pilot-scale: 130 °C (●) and 175 °C (▲). (b) Fast heating and (c) slow heating periods at lab scale (△) and pilot scale (▲) and 175 °C of working temperature.

The temperature profiles obtained along the SW treatment are plotted in Figure 2a for lab- and pilot-scale systems, and both temperatures were evaluated.

Two different heating periods were observed in both systems at 175 °C; a fast period up to 140–160 °C (Figure 2b), followed by a slower heating period until reaching the working temperature (Figure 2c). As described in Section 2.2.2, in pilot-scale SW extraction/hydrolysis, this system was preheated at 80 °C; thus, during the heating process, just 3.5 and 13.2 min were enough to reach 160 and 170 °C, respectively. However, in the lab-scale system, where water was initially at room temperature, it took 15 and 19 min, respectively, to reach these temperatures.

The heating rates for both periods were estimated according to the following equation:
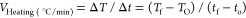
4where *T*_o_ and *T*_f_ are the initial and final temperatures in the reactor, and *t*_o_ and *t*_f_ are the initial and final times of heating, respectively, for each period.

Faster initial heating rates of 30.8 and 10.4 °C/min and final slow heating rates of 1.2 and 2.6 °C/min were obtained for the pilot- and laboratory-scale systems, respectively, when the operating temperature was 175 °C. In the pilot-scale system, the temperature remained stable during the first 40 min; however, after this period, a slight decrease was observed, and a temperature of 165.7 °C in the reactor was determined. From then, until the end of the treatment (130 min), the mean temperature was 164.6 °C. On the opposite, at the lab-scale system, the temperature ranged between 174 and 180 °C all along the experiment.

Based on the temperature profiles for both systems, it can be concluded that the heating time at the beginning of the extraction was shorter at pilot scale by preheating the equipment. However, there were problems on maintaining the working temperature by using a steam boiler as the heating system opposite to the heating jacket that allowed a more stable working temperature.

At the lowest temperature evaluated in this work (130 °C), a faster initial rate was also observed at pilot scale in comparison with lab scale because of the preheating of water; however, the temperature profiles for both scales were very similar and more stable during the SW treatment.

Because of the differences found between the different heating systems, mainly at the highest temperature evaluated, it is needed to highlight the importance of recording the temperature in order to explain the hydrolysis and degradation reactions taking place inside the reactor during SW treatment.

#### 3.2.2 Polysaccharide Fraction Extraction/Hydrolysis

**Figure 3:**
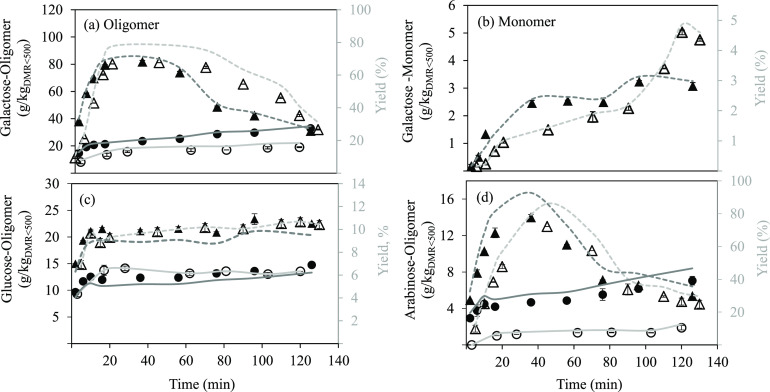
Sugar yields along SW treatment from DMR_<500_ for galactose as oligomer (a) and monomer (b), for glucose as oligomer (c), and for arabinose as oligomer (d). Principal axis, concentrations at lab scale: 130 °C (○) and 175 °C (△); pilot scale: 130 °C (●) and 175 °C (▲). Secondary axis, extraction yields at lab scale: 130 °C (—) and 175 °C (- - -); pilot scale: 130 °C (—) and 175 °C (- - -).

Figure 3a,b shows the galactose extraction yield along treatment time for both laboratory- and pilot-scale systems. Galactose was mainly released to the reaction medium as an oligomer, and small differences were observed in the extraction/hydrolysis kinetics between both scales. A faster extraction/hydrolysis was observed at pilot scale in comparison with the lab system with yield values of 60.7 and 49.9%, respectively, in the first 10 min of treatment at 175 °C. This fact could be related to the faster initial heating period followed at the pilot-scale system in comparison with the lab-scale reactor. However, the maximum oligomer yield was similar for both pilot- and lab-scale systems, 71.4% (36 min) and 78.6% (45 min), respectively, but less time was needed because of the faster heating at the pilot scale. For longer treatment times, the galactose degradation rate was higher than its formation rate by hydrolysis. The extraction yield of galactose as a monomer was very low in both designs, being only detected at 175 °C, because the hydrolysis temperature of the galactan fraction was not reached at 130 °C in both systems. As a consequence of the faster oligomer extraction at the pilot system and 175 °C, monomers appeared earlier. Nevertheless, at the end of the treatment, the monomer yield was slightly higher on the lab-scale (4.6%) than on the pilot-scale system (3.0%). These results are in concordance to the ability of water under subcritical conditions to hydrolyze solubilized galactans into galactose monomers.^20^

Much lower hydrolysis yields were obtained for glucans (Figure 3c). A similar trend was observed for laboratory- and pilot-scale systems, with maximum yields at the end of the treatment of 10.4 and 9.8%, respectively, as glucans at 175 °C because no glucose monomers were detected in the SW extracts. Moreover, an equilibrium extraction/hydrolysis yield was achieved after 20 min. No glucan degradation was observed at longer times, indicating that glucose monomers were probably not formed.

Mohan et al.^21^ proved that high temperatures are needed to hydrolyze the cellulose fraction. Below 250 °C, cellulose does not hydrolyze but dissolves, being able to produce high-degree polymerization molecules.

Regarding the arabinose fraction (Figure 3d), obtained as an oligomer, an extraction/hydrolysis yield of almost 100% was observed for pilot-scale SW at 175 °C after 36 min of treatment, showing a faster release in comparison with the lab-scale system. In both scales, the extracted amount started to decrease rapidly after reaching the maximum yield, showing a fast degradation of the solubilized arabinans. The solubilization of arabinans at 130 °C was low, with yields lower than 50% for both configurations.

**Figure 4:**
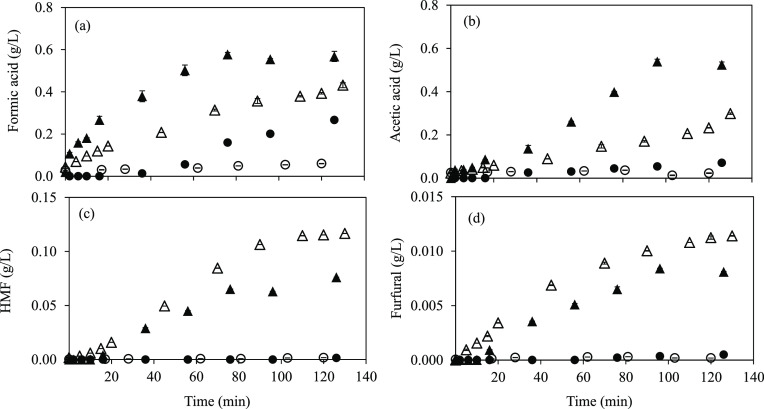
Sugar degradation compounds: (a) formic acid and (b) acetic acid; sugar dehydration compounds: (c) 5-hydroxymethylfurfural (HMF) and (d) furfural contents in SW extracts at different time intervals from DMR_<500_. Lab scale: 130 °C (○) and 175 °C (△); pilot scale: 130 °C (●) and 175 °C (▲).

The production of sugar degradation and sugar dehydration compounds is shown in Figure 4. Sugar dehydration products, such as furfural and 5-hydroxymethylfurfural (HMF), were produced in very low amounts. A notable increase in degradation product formation was observed at 175 °C after 30–40 min of treatment, coinciding with the galactan and arabinan maximums. Although this increase was a little faster on the pilot system, the final concentrations were similar for both systems: 0.57 and 0.43 g/L for formic acid and 0.52 and 0.30 g/L for acetic acid in pilot scale and lab scale, respectively. The same trend was observed for HMF and furfural formation during the SW treatment. At 130 °C, the sugar dehydration product content in the extracts was negligible and minimum for degradation compounds related to the lower amount of solubilized sugars, showing a temperature dependence of subcritical water with extraction/hydrolysis capacity. The furfural content in the extracts was very similar in both systems and lower than HMF due to the low pentose content in the raw material. Similar results were found by Jeong et al.^22^ from *G. amansis* acid hydrolysis. They observed an increase in the formic acid and HMF production at the same time that the amount of glucose in the raw material decreased.

Yoo et al.^23^ evaluated the scaling up from laboratory to pilot subcritical water systems for β-glucan hydrolysis. They found a larger extraction yield at the laboratory system (6.98%) than in pilot scale (3.01%) at 200 °C for 10 min. However, in this work, similar maximum sugar yields were achieved for both systems.

#### 3.2.3 Protein Fraction Extraction/Hydrolysis

**Figure 5:**
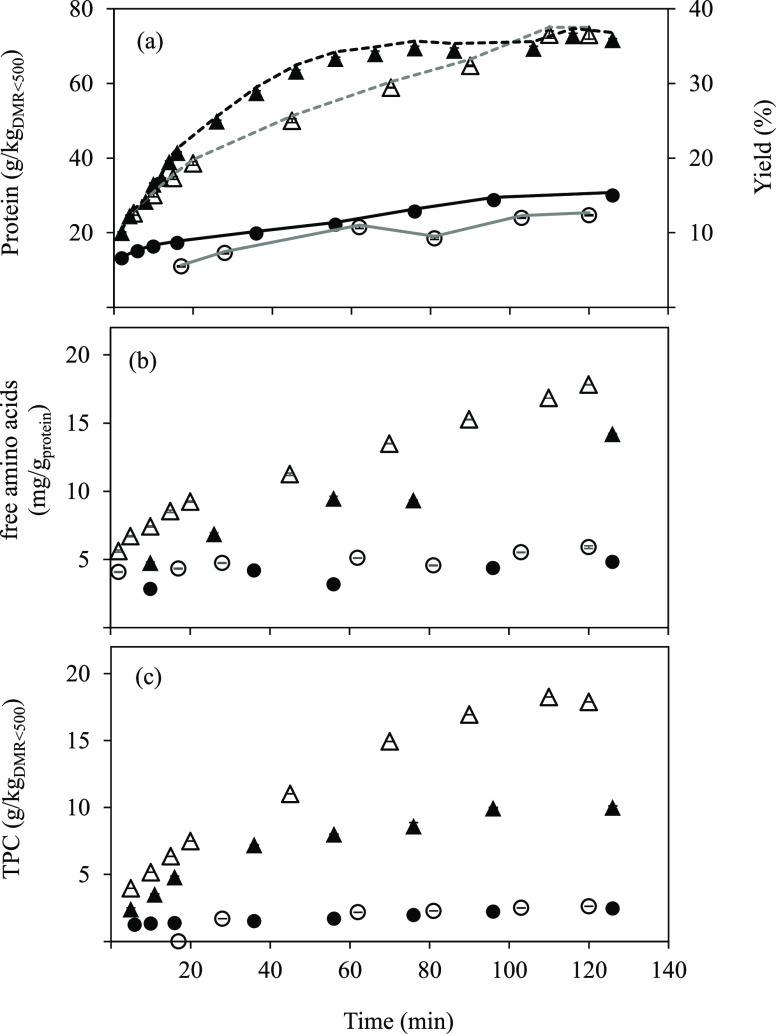
(a) Protein extraction yield, (b) free amino acids per gram of protein, and (c) TPC in SW extracts collected at different time intervals from DMR_<500_. Principal axis: concentrations at lab scale: 130 °C (○) and 175 °C (△) and pilot scale: 130 °C (●) and 175 °C (▲). Secondary axis: extraction yields for proteins at lab scale: 130 °C (—) and 175 °C (- - -) and pilot scale: 130 °C (—) and 175 °C (- - -).

Protein extraction/hydrolysis is shown in Figure 5a. Similar extraction/hydrolysis curves were obtained with both systems, although a slight faster initial extraction/hydrolysis was described for the pilot design, which could be related to the faster heating rate due to the preheating of the system. The final protein extraction/hydrolysis yield at 175 °C was 37.4 and 37.5% for pilot scale and lab scale, respectively. This fact shows the good reproducibility of the scale-up process of SW treatment on a larger scale, despite having performed only one experiment for each condition evaluated. At 130 °C, protein extraction yields were lower than 20% and very similar for both systems.

Regarding the free amino acid content, a higher production rate was observed at lab scale and 175 °C throughout the SW treatment (Figure 5b), with values of 14.2 and 17.8 mg free amino acids/g_protein_ at the pilot- and lab-scale systems, respectively. The lower yields at pilot scale could be due to the difficulty to maintain the working temperature during the process (see Figure 2a). This is explained by the fact that higher temperatures result in higher protein extraction (see Figure 5a) that can be transformed into peptide chains of different sizes, followed by the free amino acids’ release when they continue to be exposed to high temperatures because of the protein fraction hydrolysis. In this study, 175 °C was a temperature high enough to observe the protein solubilization from the raw material to the extracts and hydrolysis, being transformed into free amino acids. Also, a good correlation between lab-scale and pilot-scale SW systems was observed at 130 °C, but extracts with a lower content of free amino acids were obtained owing to a decrease in the hydrolysis capacity of subcritical water at low temperatures.

**Figure 6:**
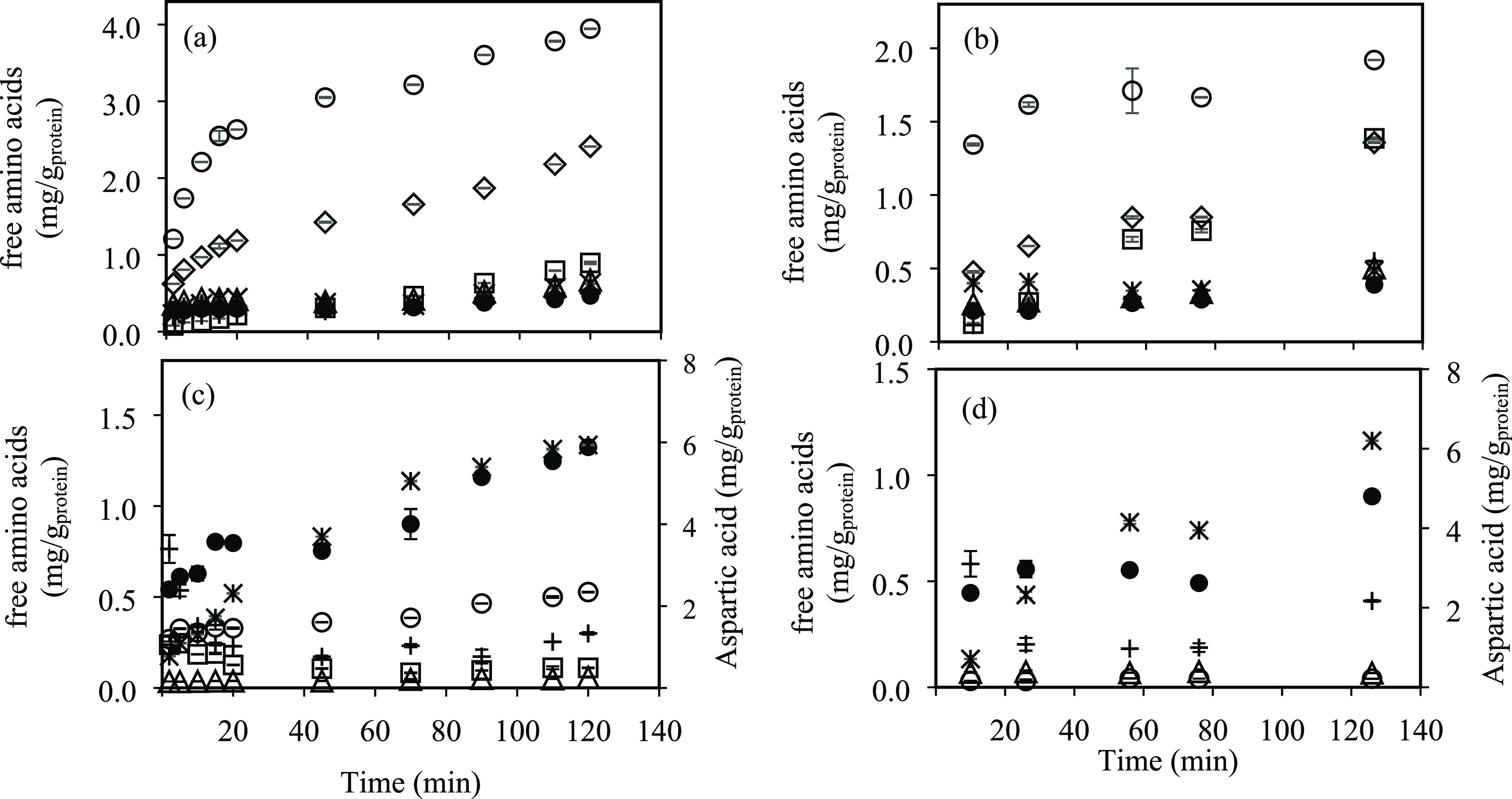
Accumulative formation of individual amino acids. Nonpolar amino acids (◇ alanine, ○ glycine, □ valine, △ leucine, + isoleucine, ∗ proline, ● phenylalanine) at (a) lab scale and at (b) pilot scale. Polar amino acids (principal axes: ○ threonine, □ lysine, △ tyrosine, + glutamic acid, ● serine; secondary axes: ∗ aspartic acid) at (c) lab scale and at (d) pilot scale. Working temperature, 175 °C (experimental data include standard deviations; *n* = 3 technical replicates).

Figure 6 shows the release curves for individual amino acids grouped into nonpolar and polar amino acids, and Table 2 lists the amino acids’ final concentrations and yields expressed as milligrams of free amino acids per milligram of amino acid in the raw material, at lab- and pilot-scale systems at 175 °C. In both systems, the greatest release of nonpolar amino acids was obtained for the smallest amino acids, with yield values of 9.6 and 4.7% for glycine and 2.6 and 1.4% for alanine, at lab and pilot systems, respectively. The concentration of the nonpolar amino acids continuously increased with increasing SW treatment time in both systems. On the contrary, the concentration of some polar amino acids such as glutamic acid and lysine was reduced as the hydrolysis process progressed, although lysine was not detected in the SW extracts from the pilot system. However, aspartic acid was one of the most produced amino acids, with 6.1 and 6.4% yields at lab scale and pilot scale, respectively.

**Table 2:** Individual Amino Acid Concentrations and Extraction Yields after SW Treatment at Lab Scale and Pilot Scale at 175 °C Working Temperature^a^

	lab-scale SWE—175 °C		pilot-scale SWE—175 °C	
	mg/g_protein_	yield (%)	mg/g_protein_	yield (%)
alanine	2.4 ± 0.04	2.6 ± 0.3	1.4 ± 0.01	1.4 ± 0.1
glycine	3.9 ± 0.1	9.6 ± 1.2	1.9 ± 0.15	4.7 ± 0.8
valine*	0.90 ± 0.05	1.3 ± 0.2	1.4 ± 0.03	2.1 ± 0.3
leucine*	0.67 ± 0.04	0.86 ± 0.15	0.50 ± 0.01	0.65 ± 0.07
isoleucine*	0.54 ± 0.03	1.2 ± 0.2	0.55 ± 0.00	1.2 ± 0.1
proline	0.62 ± 0.04	0.85 ± 0.11	0.49 ± 0.00	0.66 ± 0.04
phenylalanine*	0.47 ± 0.05	0.9 ± 0.2	0.39 ± 0.02	0.77 ± 0.12
threonine*	0.53 ± 0.07	1.5 ± 0.3	0.04 ± 0.01	0.11 ± 0.03
lysine*	0.11 ± 0.09	0.19 ± 0.18		
histidine*				
tyrosine	0.06 ± 0.01	0.17 ± 0.05	0.07 ± 0.01	0.19 ± 0.05
glutamic acid	0.30 ± 0.10	0.38 ± 0.16	0.41 ± 0.07	0.52 ± 0.12
aspartic acid	5.9 ± 0.1	6.1 ± 0.6	6.20 ± 0.09	6.4 ± 0.6
methionine*				
serine	1.3 ± 0.1	3.5 ± 0.9	0.90 ± 0.05	2.4 ± 0.5
tryptophan*				
essential amino acids (*)	3.2 ± 0.1	0.90 ± 0.14	2.9 ± 0.04	0.80 ± 0.10
total amino acids	17.8 ± 0.3	2.3 ± 0.1	14.2 ± 0.2	1.8 ± 0.1

a *n* = 3 technical replicates.

A continuous decrease in lysine content from 0.27 to 0.11 mg/g_protein_, more than 50% after 100 min of treatment, is observed in Figure 6c at the lab-scale system. Similar results were found in a previous work by using a semicontinuous SW lab system, where the selectivity toward nonpolar amino acids increased with increasing time and temperature.^1^ Rogalinski et al.^24^ also reported a high stability of alanine and glycine at subcritical water conditions, whereas lysine and other polar amino acids usually participate in Maillard reactions with reducing sugars under subcritical conditions,^6^ which could explain the decreasing content of these amino acids found in the extracts.

#### 3.2.4 Total Phenolic Content in SW Extracts

TPC determined along SW treatment is shown in Figure 5c. At lab scale, a maximum TPC of 17.9 g/kg_DMR_ was achieved at the highest temperature evaluated, while this value decreased down to 10.0 g/kg_DMR_ at the pilot plant scale; moreover, the initial rate of TPC release in lab scale was faster in comparison with that of the pilot system. However, similar extraction/hydrolysis curves and TPC were obtained at 130 °C for both systems. The lower value of TPC reached at pilot scale at 175 °C could be attributed to the decrease in the operating temperature down to 163 °C, 12 °C lower than the values maintained at the lab-scale SW reactor by using the heating jacket (Figure 2a). It is well documented that Maillard and caramelization reactions can be produced under intense heating conditions in SW treatment between reducing sugars and free amino acids such as lysine and arginine. Hence, the higher temperature after 40–60 min at lab scale could induce Maillard and caramelization reactions, whose products are well known to interfere in the TPC analysis by the Folin–Ciocalteu assay.^6^

Brown color development is an easy indicator of Maillard reaction occurrence, the brown color intensity being directly proportional to the extent of these reactions.^25^ In Figure S3, it can be observed how the color intensity in lab-scale SW extracts obtained at 175 °C progressively increased with the treatment time toward dark brown color, proving the occurrence of the Maillard reaction. Moreover, the treatment time at which the maximum brown color development was reached agreed with the maximum HMF formation and lysine disappearance, which suggests the advancement in the development of the Maillard reaction with extraction/hydrolysis time at this work temperature. However, the extracts obtained at 130 °C showed a light yellow color, and no browning was experienced throughout the extraction, agreeing with the lower content of TPC in the extracts. This fact is consistent with the lower development of Maillard reactions at low temperatures. He et al.^26^ evaluated the TPC formation under subcritical conditions. They found that the increase of time and temperature from 80 to 220 °C resulted in the increasing TPC and brown color intensity, agreeing with the high concentrations of 5-HMF in the extracts.

#### 3.2.5 Solid Residue

The solid residues after SW treatment were analyzed to determine their elemental composition, as listed in Table 3. This table also lists the elemental composition of the sieved algal residue used for SW extraction (DMR_<500_) and the original algal residue before separation by particle size (DMR). The sulfur content decreased for both SW systems as a consequence of the partial extraction of the residual agar present in the algal residue at high temperatures. A lower hydrogen content was clearly observed in the lab-scale residues as a result of the greater extraction of biocompounds during SW treatment; consequently, a lower H:C molar ratio was obtained. Also, high values for ashes (>15%) and HHV (>15,000 kJ/kg) remained in the residues evaluated after SW treatment, which could be useful to evaluate the potential of these residues to be used as fertilizers or for biofuel production. Alonso-Riaño et al.^27^ found that all the solids obtained after subcritical water extraction from brewers’ spent grain showed higher HHV than the raw material, and this value increased by increasing the working temperature. Also, Reza et al.^28^ discovered that by increasing the hydrothermal carbonization temperature, the carbon content of the samples increased, which meant to increase the HHV. For instance, they observed a HHV increase of 54% from the treated corn stover in comparison with the original sample. In the present work, just the solid residues obtained on the pilot system were found to have higher HHV in comparison with the sample used for the extraction (DMR_<500_), which could be related with the fact that the carbon content significantly increased in the pilot-scale solid residues and with the better initial heating efficiency at this scale.

**Table 3:** Elemental Analysis, Ash Content, and Estimated Higher Heating Value (HHV) of Dried Macroalga Residue (DMR), DMR < 500 μm Size Fraction (DMR_<500_), and Solid Residues after SW Treatment at Lab Scale and Pilot Scale, Expressed as % (w/w) ± Standard Deviation^a^

sample	C (%)	N (%)	H (%)	S (%)	O (%)	ashes (%)	H:C	O:C	N:C	HHV (kJ/kg)
DMR	36 ± 1^bc^	4.2 ± 0.4^c^	5.9 ± 0.2^e^	0.21 ± 0.05^c^	32.3 ± 0.3^e^	21.8 ± 1.1^d^	2.0 ± 0.1^d^	0.68 ± 0.03^c^	0.11 ± 0.01^c^	14,987 ± 466^b^
DMR_<500_	38 ± 1^c^	3.6 ± 0.1^b^	5.6 ± 0.2^cd^	0.70 ± 0.05^d^	27.2 ± 1.4^c^	24.9 ± 1.0^e^	1.8 ± 0.2^cd^	0.54 ± 0.09^b^	0.08 ± 0.02^b^	15,789 ± 250^d^
lab 130 °C	34.8 ± 1.8^b^	3.8 ± 0.3^bc^	4.7 ± 0.3^b^	0.23 ± 0.04^c^	38 ± 2^f^	17.8 ± 1.3^b^	1.6 ± 0.1^bc^	0.83 ± 0.16^d^	0.09 ± 0.01^b^	15,217 ± 618^c^
lab 175 °C	36 ± 1^bc^	3.5 ± 0.1^b^	4.6 ± 0.2^b^	0.09 ± 0.08^b^	23.1 ± 1.4^b^	32.9 ± 0.7^f^	1.5 ± 0.1^b^	0.5 ± 0.1^b^	0.08 ± 0.02^b^	15,476 ± 318^cd^
pilot 130 °C	41.7 ± 0.9^d^	3.9 ± 0.1^bc^	5.5 ± 0.1^c^	0.20 ± 0.03^c^	28.5 ± 0.9^c^	20.2 ± 0.2^c^	1.6 ± 0.1^bc^	0.51 ± 0.07^b^	0.08 ± 0.01^b^	17,087 ± 200^e^
pilot 175 °C	40.7 ± 0.2^d^	4.2 ± 0.1^c^	5.80 ± 0.03^de^	0.22 ± 0.01^c^	30.4 ± 0.2^d^	18.7 ± 0.1^b^	1.7 ± 0.1^bc^	0.56 ± 0.01^b^	0.09 ± 0.01^b^	16,877 ± 38^e^

a Values with different letters in each row are significantly different when applying the LSD method at *p* value ≤0.05 (C, carbon; H, hydrogen; N, nitrogen; S, sulfur; and O, oxygen (*n* = 3 technical replicates) (oxygen content was estimated by the difference).

### 3.3 Feasibility of the Pilot Plan Equipment

As seen in the previous section, the trends of the extraction curves for the different components evaluated were very similar when the laboratory-scale and pilot-scale systems were compared. In addition, the extraction yields achieved were high, even completely recovering some of the compounds present in the biomass used as feed sample. For this reason, the feasibility of the pilot-scale SW system by using a higher loading biomass was essayed in order to evaluate the improvement in the extraction process efficiency.

For this purpose, two different biomass loadings were evaluated: 5 and 15% (w/v) at 175 °C of working temperature for a total treatment time of 76 min. This time was selected by observing the results obtained from the extraction curves for the compounds analyzed. By the time of 76 min of extraction, it was possible to extract more than 50% for galactans, arabinans, and free amino acids, and more than 85% for glucans, proteins, and TPC, with respect to the total amount extracted throughout the extraction (see Figures 3 and 5).

#### 3.3.1 Heating Rate

**Figure 7:**
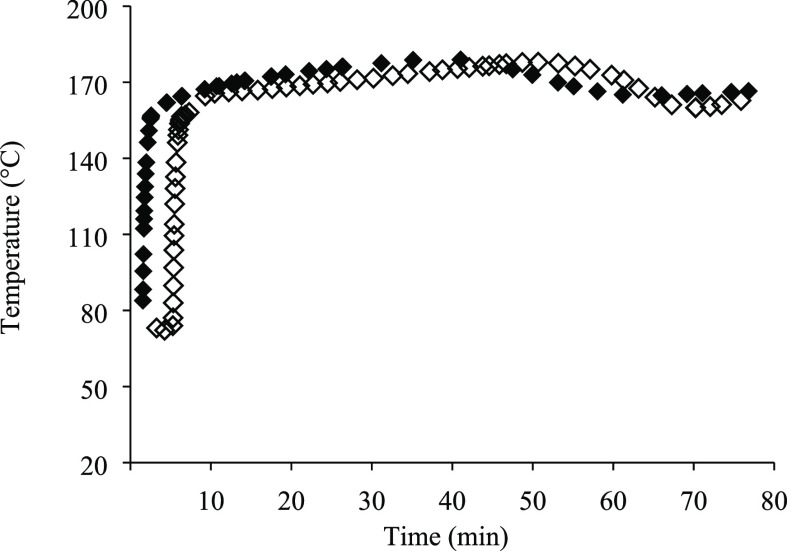
Extraction temperature profiles along SW treatment at pilot scale at 5% (◆) and 15% (◇) of biomass loadings. Working temperature: 175 °C.

The temperature profiles throughout the SW treatment at pilot scale for different biomass loadings are shown in Figure 7. A slight decrease in the temperature of the preheated water entering the reactor when using 15% of biomass loading was observed at the beginning of the extraction as a consequence of a greater amount of solid matter in the reactor; however, just 2.7 and 3.3 more minutes were necessary to reach 140 and 160 °C inside the reactor, respectively, in comparison with the experience at 5% biomass loading. Nevertheless, the same fast heating period at the beginning of the treatment, the stabilization of the temperature during the first 40–50 min, and a slight drop in temperature at the end of the treatment were observed for both conditions, with the final temperatures of 162.8 and 166.5 °C at 76 min for 15 and 5% biomass loadings, respectively.

#### 3.3.2 Bioactive Compound Recovery in the SW Extracts

**Figure 8:**
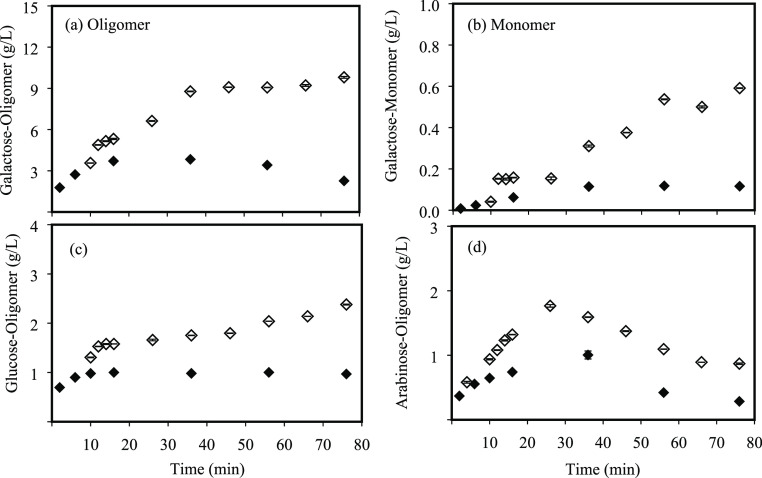
Sugars in SW extracts obtained at pilot scale at different time intervals from DMR_<500_. Galactose as oligomer (a) and monomer (b), glucose as oligomer, (c) and arabinose as oligomer (d). Concentrations at 5% (◆) and 15% (◇) of biomass loading. Working temperature: 175 °C.

The polysaccharide fraction of the extraction/hydrolysis is shown in Figure 8. A similar initial extraction was observed for oligomers when working with both biomass loadings; however, after 10 min of treatment, the extraction was greater when working at the highest biomass loading (15%) for all the sugars determined. The extraction of galactose as a monomer was very low, and yields were lower than 6% in both conditions.

**Figure 9:**
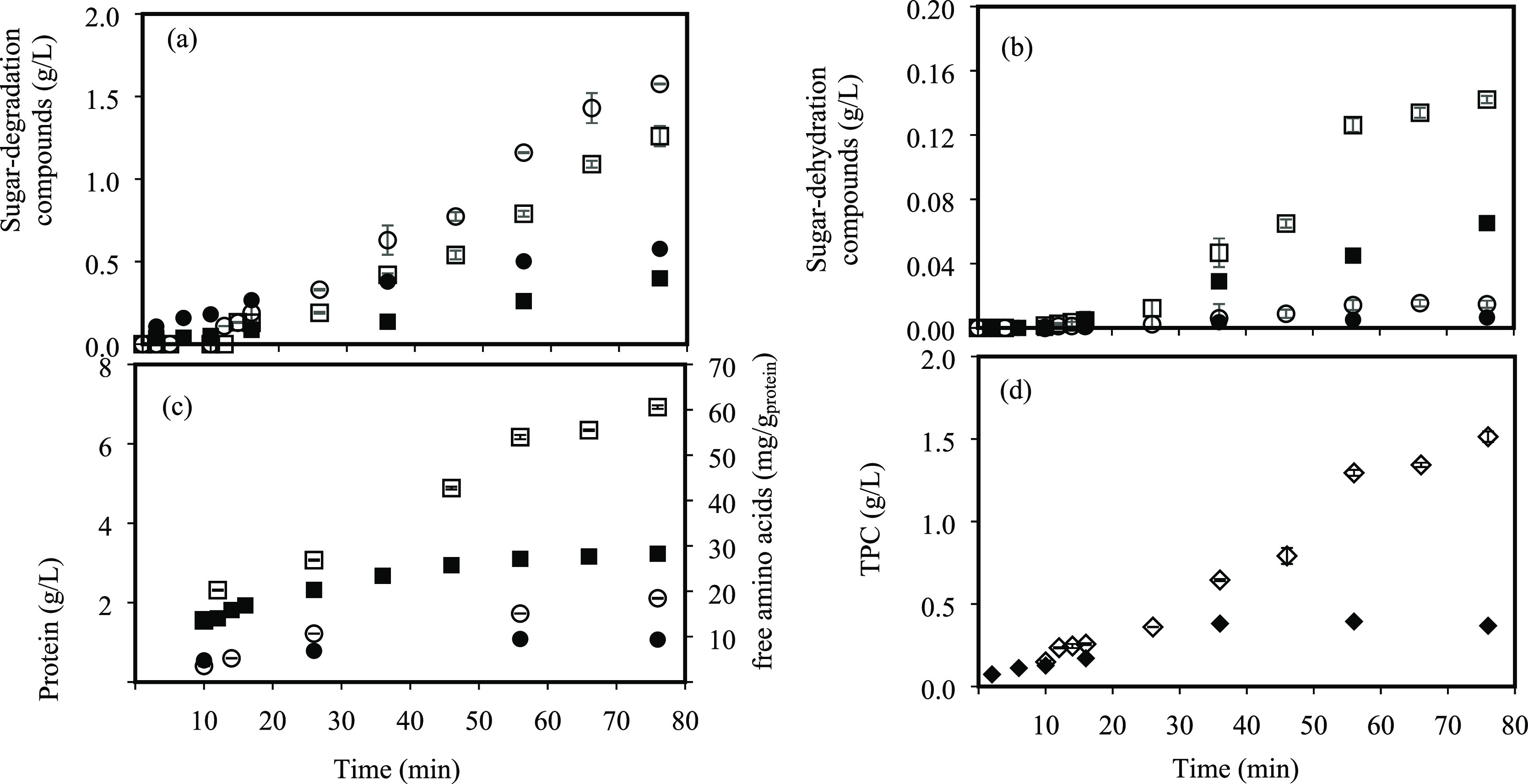
(a) Sugar degradation compounds: formic (●, ○) and acetic acid (■, □), (b) sugar dehydration compounds: furfural (●, ○) and 5-hydroxymethylfurfural (HMF) (■, □), (c) proteins (■, □) (principal axis) and free amino acids (●, ○) (secondary axis), and (d) TPC in SW extracts obtained at pilot scale at different time intervals from DMR_<500_. Filled and empty symbols are for 5 and 15% of biomass loading, respectively. Working temperature: 175 °C.

More sugar degradation compounds and HMF were produced when 15% of biomass was used (Figure 9a,b), unlike furfural, which was produced in similar and less amounts for both biomass proportions. This fact is related to the higher content of sugars found in the extracts obtained from 15% of biomass, and it could be due to the fact that when the biomass-to-solvent ratio is higher, the hydrolysis rate is faster than the extraction rate with a greater production of sugar-derived compounds. The greatest increase in this product generation occurred after 30–40 min of extraction, when the concentration of galactans and arabinans started to decrease, as it was seen before.

Regarding the nitrogen fraction, the extraction/hydrolysis of proteins and free amino acids is shown in Figure 9c. As observed for the polysaccharide fraction, the protein content in the extracts obtained by using 15% biomass load was higher than that by using a lower biomass content (5%), with the final extraction concentration values of 6.9 and 3.2 g/L, respectively. A greater hydrolysis of the protein fraction during SW treatment was also observed when working at higher biomass loadings: a slight increase in the free amino acid content was observed when 15% of biomass was used (18.5 mg/g_protein_) in comparison with 5% (9.3 mg/g_protein_).

TPC followed a similar trend: the initial extraction rates for both biomass loadings were alike, but higher extraction was observed after 30 min of SW treatment when working at a greater biomass loading, with the final concentrations of TPC of 1.5 and 0.37 g/L for 15 and 5% of biomass loadings, respectively (Figure 9d).

For all the above, the present work represents an advance in the scale-up study of SW treatment at the industrial level because the biomass proportions used both in laboratory- and in pilot-scale reactors are usually lower than those evaluated in this work. For example, Ko et al.^29,30^ studied the bioactive compound recovery from different vegetal sources by a static SW system, and both in lab scale and pilot scale, the biomass loading used was lower than 5%. Also, a study about the conversion of lignocellulose from 5% of wheat straw in water through SW treatment has been recently published.^31^ Finally, Ko et al.^32^ studied the extraction of flavonoids from a vegetal residue by using subcritical water at lab scale and pilot scale, and in both cases, the biomass loading was 4.5%.

All the above demonstrate the ability of the designed pilot-scale SW system to handle high proportions of biomass loading with a good performance and very good results in terms of biocompound concentration and extraction yields.

## 4 Conclusions

Subcritical water extraction/hydrolysis has been proven to be an efficient technology for the recovery of bioactive compounds such as carbohydrates, proteins, and free amino acids from a red algal residue. The scaling-up process from the laboratory to pilot level was performed satisfactorily, obtaining good and reproducible results in the extraction of the different compounds analyzed. Maximum extraction yields of 78.6 and 71.4% for galactans, and 86.1 and 92.7% for arabinans, at 175 °C were obtained at lab scale (45 min) and pilot scale (36 min), respectively. For glucans, a plateau phase was observed after 20 min of extraction in both systems. However, it was necessary to complete the extraction (130 min) to obtain the maximum yield for proteins, 37.5 and 37.4%, at lab scale and pilot scale, respectively. Lower yields but reproducible results were observed at 130 °C in both scales. Moreover, similar trends but higher contents of the compounds analyzed were obtained when using a higher biomass loading (15%), demonstrating the ability of the designed pilot-scale SW system to handle the high proportions of biomass in the reactor. Therefore, the feasibility of industrial-scale subcritical water treatment process through scaling up from the lab to the pilot system has been demonstrated. However, not so good correlation was observed for the extraction of TPC because of the difficulty to maintain the temperature until the end of the treatment in the pilot reactor. Temperature profile along the subcritical water treatment and extraction time resulted to be the most influential parameter because extrapolated results have been obtained despite using different heating and homogenization systems during the scaling-up study for both scales. Hence, future research about an adequate heating system which allows maintaining the temperature throughout the total extraction process in SW treatment at industrial plants is needed.

## Supporting Information Available

Particle size distribution of dried macroalgal residue; scheme of the subcritical water equipment used in lab-scale and pilot-scale systems; and photographs of the liquid extracts collected at different time intervals after subcritical water treatment at lab scale and two different temperatures, 175 and 130 °C (PDF)
