# Supplementary material for: Subcritical Water Treatment for Valorization of the Red Algae Residue after Agar Extraction: Scale-Up from Laboratory to Pilot Plant
Source: Ind Eng Chem Res. 2023 Feb 16;62(8):3503–14. doi: 10.1021/acs.iecr.2c04132 (PMC9983305; doi:10.1021/acs.iecr.2c04132)
Supplement: Supplementary file 1 — ie2c04132_si_001.pdf [file ie2c04132_si_001.pdf]

Supporting Information. Subcritical water treatment for valorization of the red algae residue after agar extraction. Scale-up from laboratory to pilot plant.

Esther Trigueros*, Cipriano Ramos, Patricia Alonso-Riaño, Sagrario Beltrán and María Teresa Sanz

Department of Biotechnology and Food Science, Chemical Engineering Division, University of Burgos, Plza. Misael Bañuelos s/n 09001 Burgos, Spain.

*_Corresponding author:_ Esther Trigueros, e-mail address: etrigueros@ubu.es

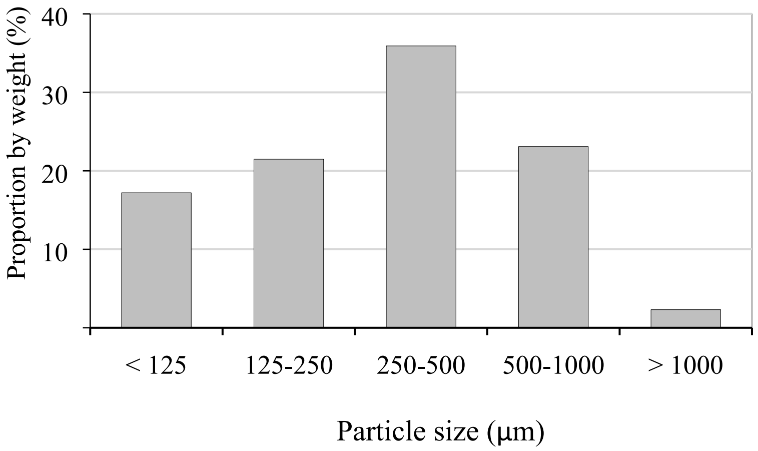

Figure S1. Particle-size distribution from dried macroalga residue (DMR).

(a) (b)

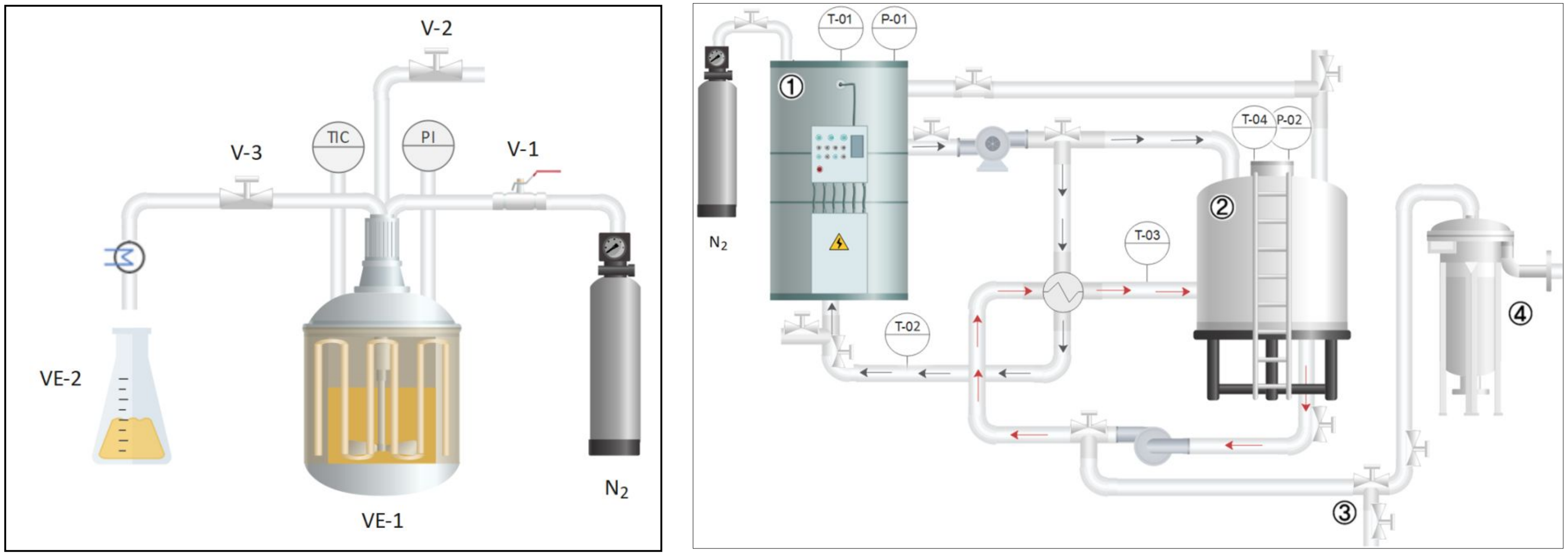

Figure S2. (a) Scheme of the lab-scale SW equipment. VE-1: reactor (0.5 L); VE-2: sample collector; V-1: pressurization valve; V-2: pressure relief valve; V-3: needle valve. **(b)** Diagram of the pilot-scale subcritical water plant designed and built at Hiperbaric's facilities. 1: steam boiler preheater and water tank; 2: reactor (25 L); 3: liquid extracts collector; 4: filtration tank.

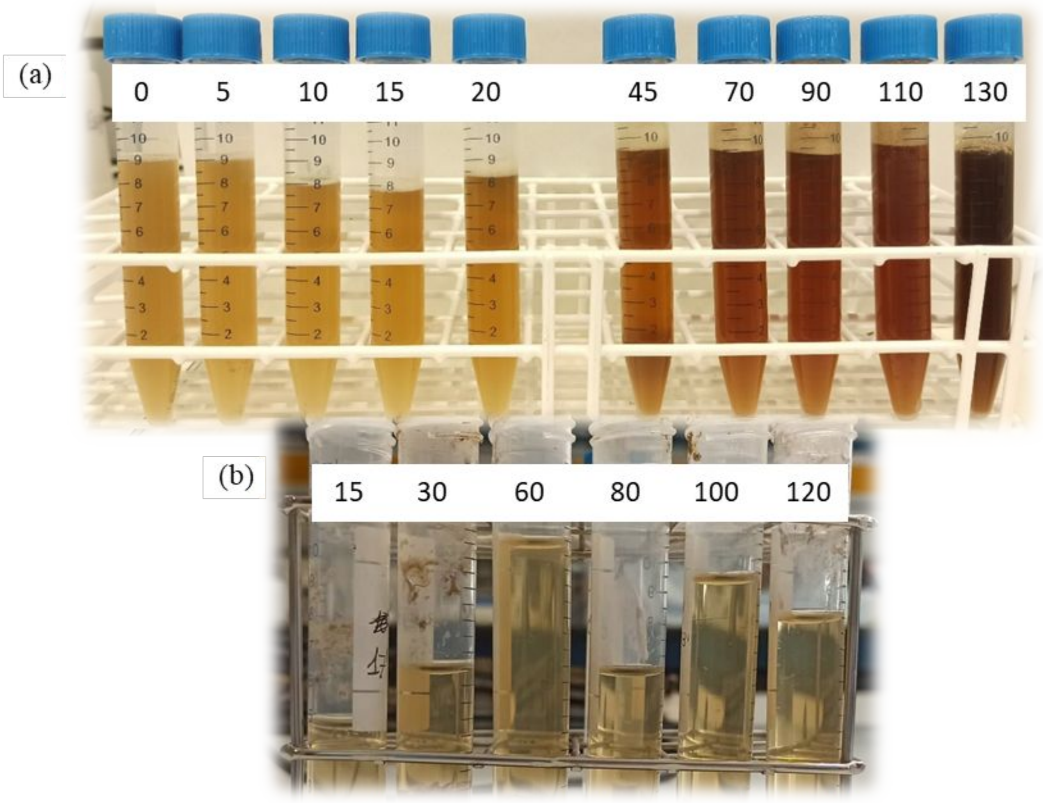

Figure S3. Liquid extracts collected at the different time intervals from $DMR_{<500}$ after subcritical water treatment at lab-scale at 175 ºC **(a)** and 130 ºC **(b)** for colour observation. (Numbers above the tubes indicate the minute at which they were collected).
